# Complete mitochondrial genome and phylogenetic analysis of *Stonogobiops yasha* (Perciformes, Gobiidae)

**DOI:** 10.1080/23802359.2019.1664949

**Published:** 2019-09-17

**Authors:** Hengtong Qiu, Cuili Wang

**Affiliations:** a Key Laboratory of Environment Change and Resources Use in Beibu Gulf, Ministry of Education, Nanning Normal University, Nanning, China;; b Guangxi Key Laboratory of Earth Surface Processes and Intelligent Simulation, Nanning Normal University, Nanning, China

**Keywords:** *Stonogobiops yasha*, mitogenome, Gobiidae, phylogenetic analysis

## Abstract

The complete mitochondrial genome sequence of *Stonogobiops yasha* was first determined in this study. The circle genome was 16,566 bp long and consisted of 13 protein-coding genes, 2 ribosomal RNA genes, 22 transfer RNA genes, and 1 control region. The mitochondrial gene arrangement of *S*. *yasha* is similar to those of most other gobies. The phylogenetic analysis using the neighbor-joining method showed that the kinship between *Stonogobiops* and *Acentrogobius* is closer than those between *Stonogobiops* and other selected genera. This is the first record of the complete mitogenome for the genus *Stonogobiops*.

*Stonogobiops yasha*, which belongs to Gobiidae, Gobioidei, and Perciformes in taxonomy, is widely distributed in reefs across the western Pacific Ocean (Yoshino and Shimada 2001). Because of their white body with reddish-orange longitudinal stripes, *S. yasha* is one of the most attractive gobies available to marine aquarists (Chang et al. 2009). We expect the establishment of *S. yasha* mitogenome could provide help for phylogenetic assessment and population studies.

The samples of *S. yasha* were imported from Manado, North Sulawesi, Indonesia (1°30′ N, 124°58′ E) to Nanning Normal University, and their genomic DNA were extracted from muscle using the standard phenol/chloroform protocol (Sambrook and Russell 2001). We designed 10 pairs of primers for sequencing through the alignment of 80 Gobiidae mitogenomes sequenced in their entirety using the primer 5 software (Singh et al. 1998). The specimen of *S. yasha* is stored at Museum of Biology, Nanning Normal University with accession no. NNNU001812.

The complete mitochondrial genome sequence of *S. yasha* has been deposited in GenBank with accession no. MN067902. The circular genome (16,566 bp) comprised 13 protein-coding genes, 2 rRNA genes (12S rRNA and 16S rRNA), 22 transfer RNA (tRNA) genes, and 1 control region. The nucleotide composition of the heavy strand of *S*. *yasha* was 28.18% for A, 27.73% for C, 16.76% for G, and 27.33% for T, with a slight A + T bias of 55.51%. On one hand, all the protein-coding genes were initiated with ATG codon, except for the *COI*, which started with GTG. On the other hand, seven protein-coding genes (*ND1*, *COXI*, *ATP8*, *ATP6*, *ND4L*, *ND5*, and *ND6*) employed the typical termination codon TAA. *ND2* and *ND3* are terminated with TAG, and the remaining protein-coding genes (*COXII*, *COXIII*, *ND4*, and *CYTB*) used one incomplete stop codon (T-). The tRNA genes were identified by the online software tRNAScan-SE1.21 (Lowe and Eddy 1997), 22 tRNA genes were found, and the length of the 22 tRNA genes varied from 65 to 75 bp. Unlike other tRNA genes, distributed on the heavy strand, the eight tRNA genes (*tRNA^Gln^*, *tRNA^Ala^*, *tRNA^Asn^*, *tRNA^Cys^*, *tRNA^Tyr^*, *tRNA^Ser^*, *tRNA^Glu^*, and *tRNA^Pro^*) were distributed on the light strand. The two ribosomal RNA genes, *12S rRNA* gene (945 bp) and *16S rRNA* gene (1684 bp), were located between *tRNA^Phe^* and *tRNA^Leu^* and separated by *tRNA^Val^*. The control region was located between *tRNA^Pro^* and *tRNA^Phe^* and consisted of 914 nucleotides.

The complete mitogenome sequences of 15 fishes and 3 mammals have been used to construct a phylogenetic tree (Figure 1) by neighbor-joining method (1000 bootstrap replicates, MEGA7 software) (Kumar et al. 2016). All the fishes were clustered into one group, while all the mammals formed the other group. The fishes in the suborder Gobioidei were grouped together while *Danio rerio* formed a side branch. In the Gobioidei, all the fishes were separated into two branches, one comprising the family Gobiidae and the other comprising the family Odontobutidae. *Stonogobiops yasha* was clustered into one clade with other two species from the genus *Acentrogobius*, which was then grouped with *Glossogobius*, *Periophthalmus*, *Gillichthys*, and *Mugilogobius* to form a cluster. This indicates that the kinship between *Stonogobiops* and *Acentrogobius* is closer than those between *Stonogobiops* and other selected genera.

**Figure 1.**
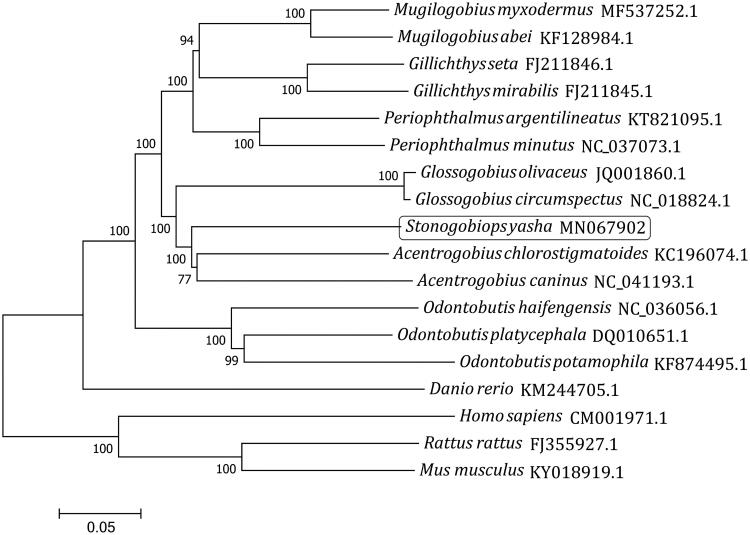
Phylogenetic analysis of 18 complete mitogenome sequences using MEGA 7 by the Neighbor-joining method and 1000 replications of bootstrap. The mitogenome sequence of *S*. *yasha* is highlighted within a box.
